# Coaches’ Emotional Intelligence and Reactive Behaviors in Soccer Matches: Mediating Effects of Coach Efficacy Beliefs

**DOI:** 10.3389/fpsyg.2019.01629

**Published:** 2019-07-10

**Authors:** Pedro Teques, Daniel Duarte, João Viana

**Affiliations:** ^1^N2i, Polytechnic Institute of Maia, Maia, Portugal; ^2^CIPER, Faculty of Human Kinetics, University of Lisbon, Lisbon, Portugal; ^3^Research Center in Sports Sciences, Health Sciences and Human Development, CIDESD, University Institute of Maia, ISMAI, Maia, Portugal

**Keywords:** coaching behaviors, coaching efficacy, emotional intelligence, multimethod research, motivation efficacy, soccer, structural equation modeling

## Abstract

In the last 10 years, emotional intelligence (EI) has become a current issue of research in psychology, and there are indicators to consider that EI should be analyzed to help the coach to behave effectively during competitions. According to Boardley’s (2018) revised model of coaching efficacy, coaches’ EI is predictive of their efficacy beliefs, which, in turn, is predictive of coaching behavior. However, little is known about the mediating effects of coaching efficacy dimensions on the relationships between coach’s EI and reactive behaviors in competitive settings. Thus, the purpose of this study is to examine mediating effects of coaching efficacy dimensions on the relationship between EI and coaches’ reactive behaviors during a game using a multimethod approach. Participants were 258 coaches of youth football players aged 9 to 17 years old. Observations *in situ* using Coaching Behavior Assessment System (CBAS) were carried on 258 football games during two seasons. At the end of each game, coaches completed the Wong and Law Emotional Intelligence Scale (WLEIS) and the Coaching Efficacy Scale (CES). Structural equation modeling (SEM) analyses revealed that motivation efficacy and character building mediated the relationship between regulation of emotion and positive and negative coaches’ reactions during game. Specifically, motivation efficacy mediated the association between regulation of emotion and positive coaches’ reactions, and the relationship between regulation of emotion and negative coaches’ reactions were mediated by motivation efficacy and character building. In addition, coaching level moderated the relationships between EI, self-efficacy and coaches’ reactive behaviors. Findings of the present study showed that coaching efficacy dimensions (i.e., motivation efficacy and character building) that have the capacity to influence their confidence in ability to affect the psychological mood and positive attitude of athletes, transfer the effects of EI (i.e., regulation of emotion) on coaches’ verbal reactions during a youth soccer game. Specifically, a coach who feels competent to regulate their own emotions would perceive high beliefs of efficacy to motivate and to build character of their athletes, and this insight has an impact on their positive verbal reactions in response to athletes’ performances.

## Introduction

Coaches exert an influential role in creating an emotional climate in youth sports (Keegan et al., 2014). This emotional climate can be provided by several coaches’ psychosocial characteristics, such as leadership styles (e.g., Price and Weiss, 2012), goal orientations (e.g., Smith et al., 2009), expectations (e.g., Coatsworth and Conroy, 2009) and coaching behaviors in competitive settings (e.g., Cumming et al., 2006). In fact, evidence suggest that coach behavior during competition influences their relationship with the athletes and their psychological development (Smith et al., 2007). Thus, the impact of coaches’ behaviors on athletes’ performance and well-being have received considerable attention in youth sport literature.

Research revealed that the interaction between behavioral, cognitive, and situational variables appear to influence coach behavior (e.g., Smoll and Smith, 1989; Chelladurai, 2007; Horn, 2008). For example, Smith et al. (1977) suggested a model that put emphasis on coach behavior toward athletes’ perceptions of those behaviors, and that these elements are influenced by three factors: coaches’ personal characteristics (e.g., coach’s personal goals, behavioral intentions, self-monitoring), athlete individual characteristics (e.g., age, perceptions of coaching norms, competition anxiety), and situational factors (e.g., competitive level, team cohesion). Smith and Smoll (1997) suggested some practical guidelines for the behavior of youth coaches, including the use of a positive coaching approach. This includes the systematic use of reinforcement in response to athletes’ effort, encouragement after failures and technical instruction given in a positive way. A negative approach based on different forms of punishment (verbal or non-verbal) to eliminate inappropriate behavior or attitudes are associated with athletes’ anxiety and motivational climate, as well as being able to provoke conflicts of interpersonal nature with the coaches (e.g., Smith et al., 2007).

Chelladurai (2007) reported that coach behavior is mostly a function of personality, expertise and experience, and will be preceded by athletes’ preferences of specific forms of behavior (e.g., instructional and guidance, social support, feedback), and situational requirements (e.g., social background of the group, goals of the group, type of task). Other researchers proposed that variables such as coaches’ emotional ability, expectancies, values, beliefs and goals are predictive of coaches’ behavior (Horn, 2008). In addition, coaches encounter a wide range of stressors (i.e., competition preparation, organizational conflicts), and must be aware of how to cope with the stressors (Olusoga et al., 2014). Hence, given that coaches establish emotional climates (i.e., the way the majority of team members feel in a particular situation) to facilitate (or detract) appropriate functioning with their athletes, manage various emotions, and have the need to improve interpersonal skills, it could be suggested that for a coach to behave effectively, they need emotional intelligence (EI) (Chan and Mallett, 2011).

Chan and Mallett (2011) argue that establishing appropriate emotional climates in teams has been associated with leaders’ EI, including the engagement in appropriate behaviors informed by the ability of the coach to perceive, use, understand, and regulate emotions (Mayer and Salovey, 1997). This conception follows the original definition of EI as a “subset of social intelligence that involves the ability to monitor one’s own and others’ feelings and emotions, to discriminate among them and to use this information to guide one’s thinking and actions” (Salovey and Mayer, 1990, p. 189). Two conceptual approaches can be identified in this field: ability EI and trait EI. The ability approach results from the contributions of Mayer and colleagues (e.g., Mayer and Salovey, 1997; Mayer et al., 2008), who emphasized a cognitive element of EI. Meta-analytic evidence advocates EI abilities as a set of progressive hierarchical specialized skills, such as to perceive emotions, to use and understand emotions, manage them and use them consistent with individual’s goals (Joseph and Newman, 2010). The hierarchical functioning and increasing complexity of these skills play an important role in facilitating individuals’ thinking and optimizing performance, thus contributing to their emotional and intellectual development. The trait approach conceives EI as a composite construct that includes individual dispositions belonging to the domain of personality and affect, but also encompasses cognitive and motivational aspects. Consequently, attributes such as stress tolerance, adaptability, impulsivity, or social competence can be found under the overarching designation of EI (Petrides et al., 2007). Following evidence of somewhat contradictory findings with trait EI (e.g., Joseph and Newman, 2010; Laborde et al., 2014b; Wagstaff and Hanton, 2016), the current study will focus the ability EI approach based on Salovey and Mayer’s framework using Wong and Law EI scale (WLEIS; Wong and Law, 2002).

In sport, EI has been linked with a wide range of outcomes among athletes, parents, and coaches (see, for a review, Meyer and Fletcher, 2007; Laborde et al., 2016; Wagstaff and Hanton, 2016). Researchers have focused on the relationship among athletes’ EI and psychosocial and behavioral correlates, including relationships with individual athletic performance (e.g., Zizzi et al., 2003; Laborde et al., 2014a), pleasant emotions that athletes experience on the day of competition, such as happiness, vigor and calmness (e.g., Lane and Wilson, 2011), use of psychological skills during and outside competitions (e.g., Lane et al., 2009), physiological responses (e.g., Laborde et al., 2011), cooperative behaviors in highly competitive conditions (Perry and Clough, 2017), and organizational functioning (e.g., Wagstaff et al., 2013). Recently, evidence showed that EI appears to play an important role in regulating parents’ sideline verbal behaviors during youth soccer games (Teques et al., 2018).

According to Hwang et al. (2013), EI is also associated with coaching efficacy. This finding suggests that coaches’ perceived ability to regulate and be aware of their own and athletes’ emotions would indicate their sense of efficacy to stimulate the performance of their athletes. Based on the original work of Bandura (1997), Feltz et al. (1999) conceived coaching efficacy as the amount to which coaches believe they have the ability to influence athletes’ learning and performance, and proposed a multidimensional model of coaching efficacy beliefs that contains four efficacy dimensions: motivation (e.g., confidence in ability to affect athletes’ psychological mood and skills), game strategy (e.g., confidence in ability to lead during the game), technique (e.g., confidence in instructional skills), and character building efficacy (e.g., confidence in ability to impact athletes’ moral attitude).

Feltz et al.’s (1999) model also suggests that coaching efficacy dimensions could be considered mediators between a variety of sources and outcomes. Empirical evidence showed that coaching experience or preparation, prior success, perceived skill of athletes and social support are positively associated with specific-dimensions of coaching efficacy (Myers et al., 2017). Nevertheless, the work of Bandura (1997) also include emotional states as a source of self-efficacy beliefs. For example, people with high levels of self-efficacy are proactive in regulating their cognitions, motivations and emotions, which allows them to take advantage of the challenges they face (Feltz et al., 2008). In this sense, Boardley (2018) developed a revised coaching efficacy model that include links between coach EI, coaching efficacy dimensions and coaching behavior. For example, Thelwell et al. (2011) showed that coaches’ emotional regulation (i.e., ability to manage their own emotions) and self-emotions appraisal (i.e., ability to evaluate their own emotions) leads to motivation and technique coaching efficacy beliefs, respectively.

Beyond sources of coaching efficacy, Feltz et al. (1999) also proposed various outcomes that should result from enhanced coaching efficacy, such as coaching behavior, player/team satisfaction, performance and efficacy. Myers et al. (2005) demonstrated the influence of perceived coaches’ engagement in efficacy-enhancing coaching behaviors and team outcome variables. Specifically, all dimensions of coaching efficacy predicted use of self-reported coaches’ engagement in coaching efficacy-enhancing behaviors (e.g., verbal persuasion, act with confidence, encourage positive talk). Sullivan and Kent (2003) also evidenced that motivation and technique efficacy were associated with athletes’ perceptions of coach leadership styles, such as perceptions of training and instruction and positive-feedback behaviors. Other studies (e.g., Sullivan et al., 2012; Hwang et al., 2013) incorporated all leadership styles (i.e., training and instruction, positive-feedback, social support, and situational consideration) demonstrating relationships with overall coaching efficacy. Altogether, these studies establish robust links between coaching efficacy and leadership styles using self-reported questionnaires. However, methodologically, what is less well-known is how coach behavior is influenced by coaching efficacy dimensions using direct observation (Boardley, 2018).

### Methodological Principles

To address this limitation in the literature, we consider a multimethod design using self-reported questionnaires about EI and coaching efficacy, and observational methods in natural settings to assess coach’s behaviors. The choice for a multimethod design was driven by the desire to have low interference on the coach’s actions in competition, observing and evaluating in natural context the genuineness of his/her behavior. Another possibility for study design could be the mixed method approach to address the research question. However, the mixed method contains qualitative and quantitative components that must be integrated to ensure the mixing of the information and would be far more elusive to assess EI, coaching efficacy, and coach’s behavior (Anguera et al., 2018).

The mixing of the data could be designed through structural equation modeling (SEM) analyses. Researchers initially used univariate and bivariate analysis to understand their data and relationships. However, to understand the complexity between variables is increasingly necessary to apply sophisticated methods of multivariate analysis, which involve the application of statistical methods (Hair et al., 2014). SEM analyses simultaneously analyze multiple variables that can represent measurements associated with latent (psychosocial) or observable (behavior). These measurements are often obtained through surveys or observations that are used to collect data.

SEM can be used to confirm *a priori* theories or identify patterns of data and relationships. Specifically, they are confirmatory in testing the hypotheses of existing and exploratory theories and concepts when they look for patterns in the data if there is little or no prior knowledge about how the variables are related. Hence, two types of SEM analyses can be performed: Covariance-based Structural Equation Modeling (CB-SEM) and Partial Least Square Structural Equation Modeling (PLS-SEM). SEM (CB-SEM) based on covariance is mainly used to confirm (or reject) theories. Specifically, a set of systematic relationships among multiple variables that can be empirically tested, determining how well a proposed theoretical model can estimate the covariance matrix for a set of sample data. In contrast, PLS-SEM, also called PLS path modeling, is mainly used to develop theories in exploratory research, using smaller samples, and is focused on explained variance in the dependent variables when examining the model (regression analysis). Although different paths can be used to test SEM models, all structural equation models are distinguished by three characteristics: (1) estimation of multiple and interrelated dependency relationships; (2) ability to represent concepts not observed in these relationships and correct measurement error in the estimation process; and (3) definition of a model to explain the whole set of relations (Hair et al., 2017). Considering the characteristics of this study that includes consistent theoretical models, a relatively large sample, and the need to test mediation between variables, CB-SEM was performed to test the study hypothesis.

### The Purpose of This Study

**FIGURE 1:**
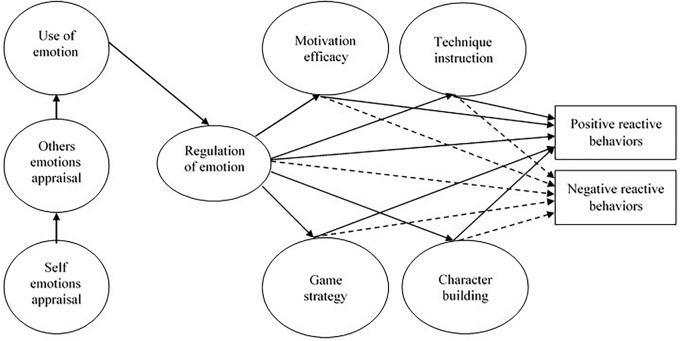
The hypothesized mediational model for the relationships between coach’s emotional intelligence (EI), coaching efficacy dimensions and reactive behaviors during soccer games. Positive paths in continuous lines; Negative paths in dashed lines.

The main purpose of this study was to examine the mediation effects of coaching efficacy beliefs on the associations between EI and reactive behaviors in soccer games. We followed Joseph and Newman’s (2010) progressive pattern of EI levels in which emotion perception, use and understanding causally precedes regulation of emotion, and Boardley’s (2018) revised model of coaching efficacy (Feltz et al., 1999, 2008), hypothesizing that coaches’ EI (i.e., regulation of emotion) will be positively associated with positive reactive behaviors and negatively associated with negative reactive behaviors (Hypothesis 1). Furthermore, we hypothesized that coaches’ EI will be associated with all coaching efficacy dimensions (i.e., motivation, game strategy, technique, and character building efficacy) (Hypothesis, 2); all coaching efficacy dimensions will positively mediate the link between EI and positive reactive behaviors (Hypothesis 3), and negatively mediate the association with negative reactive behaviors (Hypothesis 4). Finally, considering evidence of moderating effects of different levels coached (Myers et al., 2017), we were also interested in how coaching level (Hypothesis 5) moderate the links estimated in the structural model (Figure 1).

## Materials and Methods

### Participants

Participants were 258 Portuguese head coaches (*M* = 246, *F* = 12) of youth soccer players aged 9 to 17 years old. Coaches aged between 19 and 62 years old (*M* = 31.47, *SD* = 9.78); 2.7% of them had completed lower secondary education, 24.5% upper secondary education, 46.5% had an undergraduate degree, 24.1% had a master’s degree, and 2.2% had a doctorate degree. Coaches experience ranged from 1 to 33 years (*M* = 7.26, *SD* = 6.18). Coaches were involved in a long competition (soccer season) in a district standard, and coaching levels were initiation (9 – 12; *n* = 122) and specialization (13–17; *n* = 94) stages.

### Design and Procedures

Following approval from the Ethical Review Board of the Scientific Committee of the Polytechnic Institute of Maia Research Center under the reference N2I/006/04/2017, observations *in situ* were carried out in 258 soccer games over two seasons. The duration of the games were 60 min for games in the initiation stage and 80 min for games in the specialization stage, with mixed-gender participation only in the initiation stage.

#### Observer Training

Before conducting the observations of coach behaviors, a research team composed by two senior researchers and twelve undergraduate honors students were trained following the procedures of McKenzie and van der Mars (2015). The following phases were performed: (1) identification of the categories of the system (i.e., definition of behavioral categories); (2) discussion of the observation manual (i.e., team discussion about behavioral scenarios); (3) evaluation of the learning of the categories (i.e., interpretation of video clips); and (4) practice and application of the observation system *in situ*, simultaneously by all research team members. This first observation took place in three under-12 soccer games. For each of the observed categories, we found acceptable interobserver reliability values greater than or equal to 75% (Cohen’s kappa ≥ 0.75).

#### Participant Recruitment

The purpose of the study was explained to the participants and that they were observed during the game. The observers were on the field seating with adequate proximity to record coach verbal behaviors using a paper-and-pencil observation system, without giving any indication of behavior observation to avoid any socially desirable conduct. At the end of each game, coaches were approached and invited to participate in the study. Prior to the administration of the questionnaires, all participants provided signed informed consent, ensuring that they could leave the study at any time, and that the information they provide is confidential and anonymous. Then they were directed to a classroom-type setting to complete the questionnaires. Almost all coaches agreed to complete the questionnaires (99.9%). Coaches who did not agreed to participate in the study were excluded and their observational data were eliminated (*n* = 2), including only those who agreed (*n* = 258).

### Measures

#### Emotional Intelligence

The Portuguese version (Rodrigues et al., 2011) of the Wong and Law Emotional Intelligence Scale (WLEIS; Wong and Law, 2002) was used to analyze coaches’ perceptions of their EI abilities. It is a 16-item scale based on Salovey and Mayer (1990) original definition of EI: self-emotions appraisal (e.g., “I have good understanding of my own emotions”), other’s emotions appraisal (e.g., “I am a good observer of others’ emotions”), use of emotion (e.g., “I am a self-motivated person”), and regulation of emotion (e.g., “I have a good control of my own emotions”). All items were responded on 7-point Likert-type scale ranging from 1=totally disagree to 7=totally agree. Cronbach’s alpha coefficients for this study ranged between 0.77 (use of emotion) and 0.86 (regulation of emotion) for the WLEIS subscales.

#### Coaching Efficacy

The Portuguese version (Duarte et al., 2012) of the 24-item Coaching Efficacy Scale (CES; Feltz et al., 1999) was considered to examine the amount to which coaches believe they have the ability to influence athletes’ learning and performance. Participants were questioned to indicate “How confident are you in your ability to…,” and in a 5-point Likert type scale that ranges from 1=low confidence to 5=high confidence. CES measure dimensions of coaching efficacy: motivation (e.g., “build team confidence”), teaching technique (e.g., “detect skill errors”), game strategy (e.g., “recognize opposing team’s weakness during competition”), and character building efficacy (e.g., “promote good sportsmanship”). In this study, all dimensions of coaching efficacy revealed adequate Cronbach’s alpha coefficients: motivation (0.88), teaching technique (0.78), game strategy (0.86), and character building efficacy (0.74).

#### Coaching Behavior

The Coaching Behavior Assessment System (CBAS; Smith et al., 1977; Cruz et al., 2001) was used to code, record and analyze coach’s behavior during youth soccer games. CBAS examines 12 dimensions of coach behavior, categorized into two main types of coach behavior: reactive and spontaneous behaviors. Reactive behaviors are responses to positive athlete behaviors or effort and athletes’ mistakes and errors. According to Smith and Smoll (1997), coach reactive behaviors can be divided into positive and negative: positive reactive behaviors are (a) reinforcement (i.e., positive reaction by the coach to a desirable performance), (b) technical instruction (i.e., telling or showing a player who has made a mistake how to make play correctly), and (c) encouragement (i.e., encouragement to a player by a coach following a players mistake); negative reactive behaviors are (d) non-reinforcement (i.e., a failure to reinforce a positive behavior), (e) punishment (i.e., a negative response by the coach following an undesirable behavior), (f) punitive technical instruction (i.e., whenever a coach gives technical instruction in a punitive or hostile manner), and ignoring mistakes (i.e., a lack of response, either positive or negative, to a mistake on the part of a player or the team). The spontaneous categories include general technical instruction, general encouragement, organization, and general communication. For the present study, we focused on coach positive and negative elicited responses preceding athlete or team actions.

#### Data Analysis

A two-step robust maximum likelihood method of SEM approach was performed using IBM AMOS version 23 (Hair et al., 2014). First, a confirmatory factor analysis was implemented to analyze the quality of the variables adjustment to its indicators. Second, the structural model was estimated to test mediation effects, as recommended by Danner et al. (2015). Specifically, EI (i.e., self and other’s emotions’ appraisal, use of emotion, and regulation of emotion) were conceptualized to have an indirect association with coaches’ positive and negative reactive behaviors, and coaching efficacy dimensions (i.e., motivation, game strategy, technique, and character building efficacy) were considered as mediators. Bootstrap resampling procedure (1,000 bootstrap samples) with 95% bias corrected confidence intervals (CI) was used to test the significance of the direct and indirect effects. An indirect effect is considered significant (at ≤0.05) if its 95% CI does not include zero (Williams and MacKinnon, 2008). Four indexes were considered to estimate the adjustment of the model to the data (Hair et al., 2014): CFI and TLI > 0.90; RMSEA and SRMR < 0.08.

Also, a multigroup analysis was performed to examine the extent to which coaching level moderate the path coefficients assessed in the hypothesized model. Cheung and Rensvold’s (2002) criteria was considered to assess differences between models: chi-square (χ2) tests of significance and CFI difference (ΔCFI) values. The significance of the path coefficients was evaluated using critical ratio for differences produced by AMOS (significance ≥ 1.96).

## Results

A prior analysis to the data revealed 0.8% of missing data, without any missing pattern. Thus, missing data were managed using AMOS’s regression procedure. Furthermore, Mardia’s coefficient surpassed criteria (<5.0) for multivariate normality. Hereupon, following Nevitt and Hancock (2001) a Bollen-Stine bootstrap was used for further analysis. Additionally, variance inflation errors (VIF) with values ranging from 1.67 (game strategy) to 2.05 (motivation strategy) showed acceptable conditions to conduct regression analysis (VIF < 10; Hair et al., 2014).

### Measurement Model

Means, standard deviations, and bivariate correlations between study variables are presented in Table 1. Participants revealed a moderate-to-high level of self-emotions appraisal (*M* = 4.21, *SD* = 0.47), and moderate levels of game strategy efficacy (*M* = 3.33, *SD* = 0.42). Concerning coach reactive behaviors, participants expressed a mean of 49.85 (*SD* = 13.42) behaviors per game (in total 12863 coach’s reactive behaviors were recorded). Coach’s reactive behaviors were more positive (*M* = 12.64, *SD* = 6.64) than negative (*M* = 4.74, *SD* = 1.33). Regarding correlations between variables, all EI variables are associated between each other, and between all dimensions of coaching efficacy, excluding regulation of emotion with game strategy (*r* = 0.09, *p* > 0.05). In turn, regulation of emotion, motivation efficacy, character building and game strategy correlated positively with positive reactive behaviors (*r* = 0.12, *p* < 0.05; *r* = 0.17, *p* < 0.01; *r* = 0.16, *p* < 0.01; *r* = 0.21, *p* < 0.05), respectively. Also, regulation of emotion, character building and technique instruction correlated negatively with negative reactive behaviors (*r* = −0.26, *p* > 0.05; *r* = −0.22, *p* > 0.05; *r* = −0.12, *p* > 0.05), respectively.

**Table 1:** Descriptive statistics and bivariate correlations for all variables.

	1	2	3	4	5	6	7	8	9	10
1. Self-emotions app	–									
2. Others-emotions app	0.25^∗∗^	–								
3. Use of emotion	0.30^∗∗^	0.29^∗∗^	–							
4. Regulation of emotion	0.37^∗∗^	0.42^∗∗^	0.26^∗∗^	–						
5. Character building	0.28^∗∗^	0.18^∗^	0.20^∗∗^	0.31^∗∗^	–					
6. Technique instruction	0.25^∗∗^	0.32^∗∗^	0.47^∗∗^	0.18^∗∗^	0.25^∗∗^	–				
7. Game strategy	0.32^∗∗^	0.39^∗∗^	0.54^∗∗^	0.09	0.19^∗∗^	0.60^∗∗^	–			
8. Motivation efficacy	0.31^∗∗^	0.26^∗∗^	0.38^∗∗^	0.33^∗∗^	0.32^∗∗^	0.65^∗∗^	0.61^∗∗^	–		
9. Positive reactions	0.07	0.08	0.16^∗^	0.12^∗^	0.16^∗∗^	0.23^∗∗^	0.21^∗∗^	0.17^∗∗^	–	
10. Negative reactions	−0.02	−0.18^∗^	−0.13^∗^	−0.26^∗∗^	−0.22^∗∗^	−0.12^∗^	−0.18^∗∗^	−0.07	0.75^∗∗^	–
*Mean*	4.21	4.10	4.17	3.97	3.74	3.47	3.35	3.50	12.64	4.74
*Standard deviation*	0.47	0.52	0.55	0.61	0.33	0.35	0.42	0.37	6.64	1.33

*^∗^p < 0.05, ^∗∗^p < 0.01.*

The assessment of the measurement model included coach’s own and others’ emotions appraisal, use of emotion, regulation of emotion, and all coaching efficacy dimensions (i.e., motivation, teaching technique, game strategy, and character building efficacy) as latent variables. The measurement model fit to the data (χ2/*df* = 790.40 (436), *p* < 0.001, TLI = 0.939, CFI = 0.946, SRMR = 0.052, and RMSEA = 0.051; 95% CI [0.045, 0.056]).

### Structural Model

**FIGURE 2:**
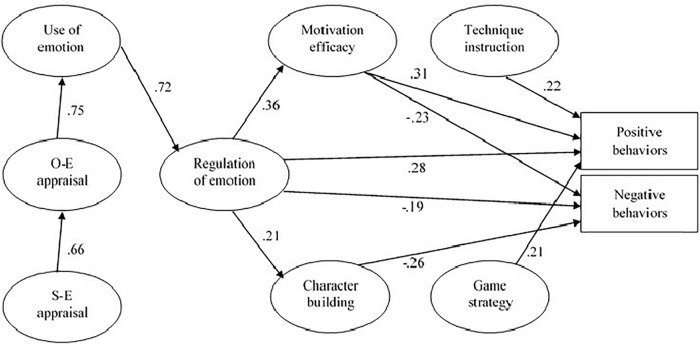
The path model. All the standardized path coefficients arc significant at the 0.05 level. Non-significant paths were excluded for visual simplicity.

The hypothesized structural model demonstrated an acceptable fit to the data (χ2/*df* = 1165.13 (512), *p* < 0.001, TLI = 0.894, CFI = 0.903, SRMR = 0.091, and RMSEA = 0.064; 95% CI [0.059, 0.068]). Some criteria had values slightly below acceptable levels (TLI < 0.90 and SRMR > 0.08); however, given that other criteria showed acceptable fit to the data (CFI > 0.90 and RMSEA < 0.08), the complexity of the model, and its theoretical adequacy to the original, we have decided to preserve the current model (Figure 2).

Figure 2 showed standardized direct effects for the hypothesized structural model. As provided, EI variables were associated in a sequential model. Moreover, regulation of emotion was associated with motivation efficacy (β = 0.36, *p* < 0.05), character building (β = 0.21, *p* < 0.05), and positive and negative reactive behaviors (β = 0.28, *p* < 0.05; β = −0.19, *p* < 0.05), respectively. Furthermore, motivation efficacy, game strategy, and technique instruction, showed significant relationships with positive reactive behaviors (β = 0.31, *p* < 0.05; β = 0.21, *p* < 0.05; β = 0.22, *p* < 0.05), respectively. Finally, motivation efficacy and character building were negatively related with negative reactive behaviors (β = −0.23, *p* < 0.05; β = −0.26, *p* < 0.05). Overall, EI and coaching efficacy dimensions account for approximately 16% of the variance of positive reactive behaviors and 21% of negative reactive behaviors (Figure 2).

#### Mediation and Moderation Analysis

Table 2 showed findings from the mediating effects between EI, coaching efficacy dimensions, and positive and negative coach’s reactive behaviors. Regulation of emotion displayed significant indirect effects on positive coaches’ reactions via motivation efficacy (β = 0.20, CI [0.12, 0.36]). Also, regulation of emotion had significant indirect effects on negative coaches’ reactions via motivation efficacy (β = −0.11, CI [−0.28, −0.06]) and character building (β = −0.35, CI [−0.35, −0.08]).

**Table 2:** Standardized indirect effects and confidence intervals.

Mediating paths	Estimate	95% CI	
		Lower	Upper
Emotion regulation → Motivation efficacy → Positive reactions	0.20	0.12	0.36
Emotion regulation → Motivation efficacy → Negative reactions	−0.11	−0.28	−0.06
Emotion regulation → Character building → Negative reactions	−0.18	−0.35	−0.08

*95% confidence intervals (CI) do not include zero for indirect effect significance.*

Another goal of the study was to analyze the moderation effects of coaching level in the hypothesized model (*n*_initiation_ = 122; *n*_specialization_ = 94). Accordingly, a multigroup confirmatory factor analysis were performed. The fit of both unconstrained [χ2/*df* = 1834.59 (1024), *p* < 0.001, TLI = 0.893, CFI = 0.904, RMSEA = 0.050 (CI = 0.046, 0.054)] and constrained structural paths [χ2/*df* = 1123.89 (1195), *p* < 0.001, TLI = 0.890, CFI = 0.898, RMSEA = 0.051 (CI = 0.048, 0.056)] models was slightly below the acceptable levels. However, non-significant χ2 statistic indicated that these models were invariant [Δχ^2^(34) = 18.33, *p* > 0.05]. Critical ratios for differences demonstrated that structural paths from regulation of emotion to positive coaches’ reactions was significantly different (*Z* = 2.44, *p* < 0.05). The path coefficient for initiation (β = 0.61, *p* < 0.01) was greater than the coefficient for specialization (β = 0.19, *p* < 0.01), indicating that initiation coached with high regulation of emotion strategies were more likely to use positive reactive behaviors than specialization coached.

## Discussion

The main purpose of this study was to examine the mediating effects of coaching efficacy dimensions on the association among coach’s EI and reactive behaviors during soccer games. Findings revealed that coaches’ regulation of emotion was positively associated with positive reactive behaviors, and negatively with negative reactive behaviors (Hypothesis 1). As high levels of coaches’ regulation of emotion are related with behaviors evidenced as positive for youth athlete’s development (e.g., Smith and Smoll, 1997), high emotion regulation coaches are likely to exhibit fewer negative behaviors (i.e., punishment, punitive technical instruction, ignoring mistakes) in reply to undesirable emotion-eliciting situations during soccer games. These promising results suggest that improvement of coaches’ regulation of emotion may have the potential to be enhanced in order to improve coaches’ desirable behaviors during athletes’ participation in competitive sport.

In line with earlier studies (Thelwell et al., 2011; Hwang et al., 2013), EI (regulation of emotion) was positively associated with coaching efficacy dimensions (Hypothesis, 2). However, this study provides more consistency than previous evidence that reported problems with the reliability of the scales (Boardley, 2018). A closer look into the methodology of the present study, suggest that the use of the SEM analysis allows to test the global adjustment of simultaneous relationships between variables, which supports Boardley’s (2018) revised coaching efficacy model, as well as the individual significance of the parameters in a single methodological framework.

Additionally, regulation of emotion was associated with coach’s reactive behaviors via coaching efficacy dimensions (i.e., motivation and character building efficacy). These results expanded previous studies demonstrating mediating effects of coaching efficacy dimensions on the link between EI and individuals’ behaviors (e.g., Hwang et al., 2013). Furthermore, as noted above, those who better regulate emotions will enhance their sense of efficacy to affect athletes’ psychological mood and positive attitude. In turn, these coaching efficacy dimensions were associated with short-term elicited responses preceding athlete or team actions. In other words, the mediating effects of coaching efficacy suggests that coaches with high emotional regulation can manage emotions effectively and are thus more likely to believe that they have the capacity to affect the learning and performance of their athletes, as well as to adopt favorable behaviors during the game.

Furthermore, the results regarding moderating effects of coaching level showed that the mediational model was invariant across initiation and specialization levels (Hypothesis 5). However, an analysis of the structural paths exposed that a path coefficient for initiation (i.e., regulation of emotion ? positive coaches’ reactions) was greater than the coefficient for specialization. While the research on potential moderators has provided inconsistent results (Myers et al., 2017), the results of the present study corroborate the idea that coaching level moderated the relationships between the proposed sources of coaching efficacy and the dimensions of coaching efficacy.

### Methodological Considerations

Researchers described the multiple types of data sources using diverse research designs – qualitative, quantitative, observational, electronic, images, and sensor data, among others – that might be used to improve the level of detail to understand behavior in sport (Anguera et al., 2018). This form of research represented methodological challenges for researchers to understand the relationships between coaches’ EI, efficacy beliefs and reactive behaviors in soccer games. These challenges included the need for extensive data collection, intensive analysis of both observational and statistical data, and the requirements that researchers be familiar with quantitative and qualitative forms of research. These methods were applied to converge data from naturalistic observation of coach behavior with data of self-reported EI and efficacy beliefs questionnaires.

The convergence of the data was performed through CB-SEM analyses to achieve the primary purpose of the study to examine the mediation effects of coaching efficacy beliefs on the relationships between EI and reactive behaviors in soccer games. Thus, CB-SEM was performed to test and confirm consistent theoretical models (e.g., EI, coaching efficacy). However, future studies may consider the alternative PLS-SEM when the sample is smaller (because is costly to have large samples combining data from systematic observations) (Hair et al., 2017).

Multivariate statistical analyses have long been used to improve the quality of research designs that use a large amount of data from surveys, and we noted how a number of studies are currently published using SEM analysis in social and sports sciences. These investigations share common features, specifically related with data from surveys (Kline, 2011). However, our approach within the present study was that information from other different sources (e.g., systematic observation, surveys) could be combined. In this study, we showed statistical methods for combining information, identifying research needs, and proposing steps that can be taken to facilitate answers for understating precursors of coach’s behavior. Future research should shift from sole reliance on surveys to a system that relies on surveys along with *in situ* observations and other research methods, making use of the strengths of each data source.

### Limitations and Future Research

The results of the present study seem to open new avenues for the exploration of a traditional theme of sports psychology, but limitations should be considered. First, due to the cross-sectional nature of the study it is difficult to establish causality of effects and thus the application of the findings to applied contexts are limited. Hence, future studies should analyze these relationships considering a season-long perspective, in order to understand for example how EI, dimensions of coaching efficacy, and coaches’ reactive behaviors reciprocally impact each other. Second, for researchers on coaching science (e.g., Smoll and Smith, 1989; Chelladurai, 2007; Horn, 2008) there are variables that influence the coach-athlete relationship, such as athlete performances, coach personality, or situational characteristics (e.g., culture, gender, age, collective/individual sports, group size). For example, personality traits have been shown to play a relevant role in the dyadic function between coach and athlete (Jackson et al., 2011). In particular, a greater dissimilarity between extraversion and openness was related with less commitment between coach and athletes. As studies in sport are rare, researchers should consider coach personality traits to understand the links between coaches’ EI, efficacy beliefs, and reactive behaviors. Also, researchers should consider that EI may be a learning process. John and Gross (2004) found that older adults were more likely to use effective emotional regulation strategies, including positive construction of emotion-eliciting events, than young adults; thus, researchers should explore how coaches’ regulation of emotion in competitive settings change depending on the age and years of coaching experience. Likewise, there is also considerable body of work pointing to cross-cultural differences in leaders’ EI. Leaders’ EI and subordinates’ performance is stronger in cultures that give priority to group membership, good work relationships with others, and tolerance toward ambiguities (Miao et al., 2018). It would be interesting to extend this study to other cultures that are likely to regulate emotions and express different coach behaviors in competitive settings. Third, from a measurement point of view, although WLEIS (Wong and Law, 2002) have presented once again good psychometric characteristics in sport (e.g., Lee and Chelladurai, 2016; Teques et al., 2018), more research is needed to develop and validate a sport-specific self-report EI ability measure. This would involve the steps commonly used to create a sport-specific scale in sport settings and an in-depth examination of the relationships between the measure and other established constructs associated with EI, such as personality or achievement motivation (Mayer et al., 2008), to clarify any redundancies often associated with EI trait approach (e.g., Joseph and Newman, 2010). Finally, the type of sport may influence the relationships between EI and efficacy beliefs (e.g., Myers et al., 2017). In this sense, future research should investigate the structural invariance of the model between the various modalities and levels of performance, trying to perceive any differences in their relationships.

## Conclusion

In synthesis, the main finding of this study revealed that coach’s motivation efficacy and character building mediated the association between regulation of emotion and positive and negative coaches’ reactions during game. From a practical standpoint, this indicates that coaches who are competent to regulate their own emotions would perceive high beliefs of efficacy to stimulate and to enhance positive attitudes of their athletes, and this is reflected in more positive (e.g., positive reactions by the coach to desirable athletes’ performances) and less negative behaviors (e.g., negative responses by the coach following undesirable athletes’ actions) during the game. Additionally, this study showed that coaching level moderated the associations between EI, efficacy beliefs and coaches’ reactive behaviors. Specifically, moderation analysis indicated that coaches of soccer teams in the initiation stage tended to feel more competent to regulate their own emotions, and this is reflected in more positive behaviors during the game, in comparison with coaches of teams in the specialization stage. Also, at an elementary descriptive level of study results, the majority of coach reactive behaviors were more positive (e.g., positive reinforcement, technical instruction, and encouragement) than negative (e.g., non-reinforcement, punishment, punitive technical instruction, and ignoring mistakes). Thus, whereas negative coach’s behaviors may be mainly concerning for sport psychologists in coach training programs (e.g., Smith et al., 2007), this study suggests that these reactive behaviors are less frequent. Finally, the findings support Boardley’s (2018) conceptual assertion that EI and coaching efficacy beliefs are determinants of coach behaviors by revealing that coaches’ EI (i.e., regulation of emotion) is linked positively with desirable coach behaviors and negatively with undesirable coach behaviors via relevant coaching efficacy dimensions (i.e., motivation and character building efficacy). In general, this study elucidates coach’s emotional experiences in their technical area during a soccer game and suggests that EI and coach’s efficacy beliefs strategies may be useful approaches to include in coach training programs.

## Data Availability

The datasets for this manuscript are not publicly available because ethical restrictions (contain information that could compromise the privacy of research participants), but are available from the corresponding author on reasonable request. Requests to access the datasets should be directed to pteques@ipmaia.pt

## Ethics Statement

Ethical approval for this study was obtained from the Scientific Committee of the Polytechnic Institute of Maia Research Center under the reference N2I/006/04/2017. All subjects gave written informed consent in accordance with the Declaration of Helsinki. The protocol was approved by the Scientific Committee of the Polytechnic Institute of Maia Research Center.

## Author Contributions

PT and DD were enrolled in study design, data collection, and writing the first draft of the manuscript. PT and JV participated in data analysis, and writing of the methodology and results. PT, DD, and JV participated in final revisions of the manuscript. All authors read and approved the final version of the manuscript and agreed with the order of presentation of the authors.

## Conflict of Interest Statement

The authors declare that the research was conducted in the absence of any commercial or financial relationships that could be construed as a potential conflict of interest.
